# Distinct metabolic syndrome profiles across Asian American subpopulations

**DOI:** 10.1038/s41598-025-15183-6

**Published:** 2025-08-12

**Authors:** Lin Zhu, Aisha Bhimla, Anjali Mann, Loretta Hsueh, Grace X. Ma

**Affiliations:** 1 Center for Asian Health, Lewis Katz School of Medicine, Temple University, 3440 N Broad Street, Ste. 320, Philadelphia, PA 19140 USA; 2 Department of Psychology, University of Illinois at Chicago, 1007 W. Harrison St, Chicago, IL 1009 BSB, 60607 USA; 3 Department of Urban Health and Population Science, Lewis Katz School of Medicine, Temple University, 3440 N Broad St., Kresge Bldg, Philadelphia, PA 19140 USA

**Keywords:** Metabolic syndrome, Asian american, Data disaggregation, Body mass index, Endocrine system and metabolic diseases, Epidemiology

## Abstract

Metabolic Syndrome (MetS) is a cluster of conditions increasing risks for cardiovascular diseases. Existing research on Asian Americans has been limited by aggregate data reporting, which masks critical intra-group health variations. In this study, we conducted a comprehensive disaggregated analysis of MetS prevalence across diverse Asian American ethnic groups, revealing nuanced health disparities that challenge monolithic health narratives. We analyzed National Health and Nutrition Examination Survey data from 2011 to 2016. The Analysis sample consisted of five Asian American ethnic groups: Chinese (*n* = 652), Asian Indian (*n* = 409), Filipino (*n* = 262), Vietnamese (*n* = 243), and Korean (*n* = 215). Logistic regression assessed associations between MetS and demographic factors. Significant variations in MetS prevalence emerged across subpopulations and sexes. For example, among women with BMI < 23, Filipino women showed significantly higher MetS prevalence (31.70%) compared to Chinese women (14.45%). Among men with BMI 23-27.4, Asian Indian men exhibited higher prevalence (50.80%) than Vietnamese men (22.66%). Our findings also shed light on the nuanced associations between modifiable lifestyle factors and MetS risks in these Asian subpopulations. This study underscores the critical importance of data disaggregation in understanding health disparities. The findings support developing targeted interventions that address the unique metabolic health profiles within Asian American communities.

**Supplementary Information:**

The online version contains supplementary material available at 10.1038/s41598-025-15183-6.

## Introduction

Metabolic Syndrome (MetS) is a cluster of related physiological, biochemical, clinical, and metabolic irregularities, which substantially increase the risk of cardiovascular diseases (CVD) and type 2 diabetes^1,2^. The burden of CVD among Asian Americans remains underexplored, despite recent national data revealing a higher burden of diabetes and hypertension in this population compared to their white counterparts^3–6^. MetS epidemiology in Asian Americans is pivotal for accurately estimating current and future CVD burden and devising targeted public health interventions^7–9^. Asian Americans constitute the fastest-growing racial group, with projections indicating continued growth through 2060. This population includes over 20 distinct ethnic groups, each with unique migration histories, cultural practices, and health profiles^10–13^.

Current scientific understanding of the MetS burden in this population has been limited by aggregate reporting of data, which mask heterogeneity in the Asian American population^4,14,15^. The “model minority” myth, widely perpetuated in both public discourse and public health research, contributes to a misleading perception of Asian Americans as a homogeneous group characterized by high financial achievements and uniformly positive health outcomes^16,17^. This stereotype has led policymakers and researchers to treat Asian American populations as monolithic, often overlooking significant intra-group differences^13,18,19^. Contrary to the model minority narrative, Asian American communities exhibit extensive diversity in income^20^, educational attainment^21^, and English proficiency^22^, as well as many aspects of sociopolitical lived experiences^23^. These variations are also reflected in health behaviors and outcomes such as lifestyle behaviors^24,25^, mental illness^22,26^, hypertension^10^, obesity^27^, and various types of cancer^28–30^. Analysis using disaggregated data is a critical first step in addressing the overlooked health and social needs of Asian American populations and challenging their continued marginalization^4,13,19^, whereas traditional MetS risk assessment may inadequately capture disease burden in these communities and masking the needs to different demographic groups.

This study examined MetS prevalence disaggregated across five Asian American subgroups, analyzing sex, BMI, sociodemographic, and lifestyle factors. Given the growing size of the Asian American population and the established link between MetS and cardiovascular mortality, using disaggregated data to understand subgroup-specific patterns is crucial for healthcare providers and policymakers. By addressing key knowledge gaps in MetS epidemiology, our findings aim to inform targeted clinical practice, public health strategies, and policies to reduce cardiovascular disparities in these communities.

## Methods

### Study sample

This cross-sectional study utilized data from non-Hispanic Asian (hereafter referred to as Asian) adults in the 2011–2016 National Health and Nutrition Examination Survey (NHANES), which included six cycles of NHANES data. NHANES is part of a series of health-related programs conducted by the Centers for Disease Control and Prevention’s National Center for Health Statistics, aimed at monitoring trends in disease prevalence and examining the relationship between diet, nutrition, and health^31^. NHANES uses a multistage, stratified design to produce a study sample that is representative of the noninstitutionalized civilian resident population in the 50 states and the District of Columbia^31^. The survey consists of two parts. First, survey questionnaires were administered to eligible participants at home, where person-level demographics, health, and nutrition information were collected. Then, participants were invited to visit specially equipped mobile examination centers for a standardized health examination. The survey procedures are detailed elsewhere^31^.

The 2011–2016 NHANES oversampled Asians and other subpopulations to enhance the precision of estimates for these groups. To support the oversampling of the Asian population, NHANES provided survey materials and a promotional video in traditional and simplified Mandarin, Korean, and Vietnamese^32^. This study utilized both public use and restricted access NHANES data. The detailed Asian ethnicity variable was available in the restricted access data. Participants were included in this study if they: (1) were aged 18 years or older, and (2) self-identified as one of five major Asian ethnic groups (Chinese, Asian Indian, Filipino, Vietnamese, or Korean) or as non-Hispanic White. Participants were excluded if they had missing data on any of the five components of MetS described in the section below. The final sample included 652 Chinese Americans, 409 Asian Indian Americans, 262 Filipino Americans, 243 Vietnamese Americans, 215 Korean Americans, and 6,318 non-Hispanic White (NHW) adults.

### Measures

Metabolic syndrome. We used the 2005 definition from the International Diabetes Federation (IDF) to define MetS^33^. Under the IDF definition (Supplementary Table 1), an individual is considered to have MetS if they have central obesity (waist circumference ≥ 90 cm for South and East Asian men, ≥ 80 cm for South and East Asian women, with ethnicity-specific values applied if BMI > 30 kg/m²), plus any two of the following four factors: (1) elevated triglycerides (≥ 150 mg/dL) or specific treatment for this lipid abnormality; (2) reduced HDL-C (< 40 mg/dL in males, < 50 mg/dL in females) or specific treatment for this lipid abnormality; (3) elevated blood pressure (≥ 130/85 mm Hg) or treatment for previously diagnosed hypertension; and (4) elevated fasting plasma glucose (≥ 100 mg/dL) or previously diagnosed T2DM. MetS was a binary measure and was categorized as those that met the criteria for MetS or did not have MetS.

#### Covariates

Body mass index (BMI). BMI is an estimated measure of body fat based on an individual’s height and weight. It is calculated as weight (kg) divided by height squared (m²), was assessed using Asian-specific World Health Organization cutoffs for overweight (≥ 23.0 kg/m²) and obesity (≥ 27.5 kg/m²).^35^ Individuals with a BMI below 23 were classified as underweight or normal weight, those with a BMI between 23 and 27.4 were categorized as overweight, and those with a BMI of 27.5 or higher were classified as obese. We did not distinguish between underweight (< 18.5) and normal weight (18.5–22.9). This approach is consistent with previous NHANES studies examining metabolic syndrome or obesity in Asian populations^35–37^, where the focus is primarily on identifying individuals at increased cardiometabolic risk (i.e., those with BMI ≥ 23) rather than distinguishing between underweight and normal weight categories.

#### Demographic characteristics

We performed the analysis separately by sex (women or men). Additionally, we controlled for age (in years), marital status (currently married or not), education level (high school or below; some college or college degree; or graduate degree), and poverty level (ratio of annual family income to the federal poverty line).

#### Modifiable lifestyle behaviors

We estimated physical activity energy expenditure with the metabolic equivalent of task (MET) minutes spent in moderate-to-vigorous physical activity (MVPA). NHANES collected data on leisure-time physical activity by intensity through questionnaires assessing vigorous recreational activities (e.g., basketball) and moderate recreational activities (e.g., swimming). Participants reported frequency (days per week) and duration (minutes per day) for each activity type. Following analytical guidelines similar to the International Physical Activity Questionnaire (IPAQ)^38^, NHANES defined vigorous physical activity as ≥ 8 METs and moderate physical activity as ≥ 4 METs. MET-minutes/week for each activity was calculated as the product of MET score and minutes per week performing the activity. Total MVPA MET-minutes/week was the sum of MET-minutes from moderate and vigorous activities. We then created four categories based on the cut-off points outlined in the 2018 Physical Activity Guidelines for Americans^39^: sedentary (individuals reporting no regular physical activity), insufficient (individuals engaging in 1–499 MET-minutes of activity per week), moderate (individuals performing 500–1,000 MET-minutes of activity per week), and high (individuals engaging in more than 1,000 MET-minutes of activity per week)^40^. This measurement for physical activity was widely used in other studies of NHANES. Tobacco use was assessed in two categories: whether an individual currently uses any tobacco products or not. Alcohol use was measured based on the amount and frequency, categorized into four groups^41^. Lifetime abstainers reported fewer than 12 drinks in their lifetime. Former drinkers had consumed more than 12 drinks in their lifetime but none in the past year. Current drinkers were divided into non-excessive (≤ 14 drinks/week for men or ≤ 7 drinks/week for women) and excessive (> 14 drinks/week for men or > 7 drinks/week for women, or consuming ≥ 5 drinks in one day at least once in the past year). These lifestyle behavior measurement approaches have been widely used in other studies utilizing NHANES data^42–45^.

### Statistical analysis

We incorporated the necessary sample weights following the guidelines set by the National Center for Health Statistics to adjust for the complex survey design and the oversampling of Asian Americans^46^. We applied the *svy* command in Stata to adjust for the sample weights. Age-adjusted and sex-specific prevalences of MetS were calculated for each BMI category. Specifically, we used the age distribution from the 2010 US Census to standardize the prevalences and means of MetS and its five components. Age-adjusted prevalence rates were expressed as percentages with 95% confidence intervals (CIs). The weighted prevalence and 95% CIs of MetS were computed separately for the five Asian American ethnic subgroups and non-Hispanic Whites (NHWs) across different BMI categories, as well as for men and women. We also conducted binary logistic regression analyses to examine the association between sociodemographic factors and three modifiable lifestyle behaviors with the presence of MetS. We reported log odds and discrete changes in predicted probabilities, representing the change in MetS risk for a one-unit increase in continuous predictors or category changes (e.g., married vs. unmarried), with other covariates held at their means. From these results, we generated sex-specific predicted probabilities of MetS across BMI categories, which were visualized in charts. A p-value of 0.05 or lower was deemed statistically significant. We interpret p-values between 0.05 and 0.10 as indicating marginal significance, consistent with practices in some areas of epidemiological research, particularly in exploratory analyses. While *p* <.05 remains the conventional threshold for statistical significance, we include these results to highlight potentially important trends warranting further investigation. All statistical analyses were performed using Stata 16^47^. Because access to data on Asian ancestry is restricted, analyses were conducted at the National Center for Health Statistics Research Data Center (NCHS-RDC) in Philadelphia, Pennsylvania following review and approval by the NCHS Research Ethics Review Board. All methods were carried out in accordance with relevant guidelines and regulations.

## Results

**Fig. 1:**
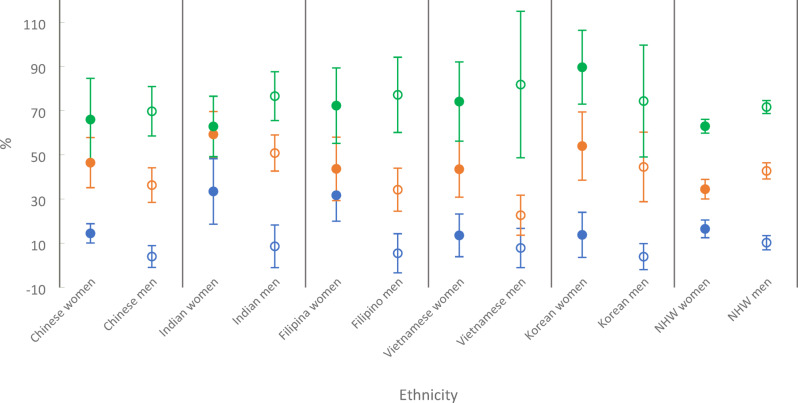
Age-Adjusted, Sex-Specific Prevalence of MetS and 95% CI at Three BMI Levels among Five Major Asian American Ethnic Groups and Non-Hispanic Whites: NHANES 2011–2016 (*N* = 8,099). Women: blue solid dot: BMI < 23; orange solid dot: BMI 23–27.4; green solid dot: BMI > = 27.5. Men: blue circle: BMI < 23; orange circle: BMI 23–27.4; green circle: BMI > = 27.5. Acronyms: MetS = metabolic syndrome; CI = confidence interval; NHW = non-Hispanic white.

Figure 1 presents the age-adjusted prevalence of MetS by sex and ethnicity. Exact values and 95% confidence intervals are provided in Supplementary Table 2. We found that there was a positive association between an increased BMI level and the prevalence of MetS within each ethnic group. In the Filipino subsample, as an example, MetS prevalence increased from 31.70% (95% CI: 19.92% − 43.47%) for those with a BMI < 23, to 72.27% (95% CI: 55.20% − 89.33%) for those with a BMI > = 27.5 among women, and from 5.44% (95% CI: −3.34% − 14.29%) for those with a BMI < 23, to 77.15% (95% CI: 60.13% − 94.17%) for those with a BMI > = 27.5 among men.

There were drastic differences in MetS rates across ethnic groups. For example, among women with a BMI < 23, the prevalence of MetS was significantly higher in Filipina women (31.70%, 95% CI: 19.92% − 43.47%) compared to Chinese women (14.45%, 95% CI: 10.08% − 18.82%). Among men with a BMI between 23 and 27.4, Asian Indian men (50.80%, 95% CI: 42.64% − 58.96%) exhibited a significantly higher prevalence of MetS compared to Vietnamese men (22.66%, 95% CI: 13.60% − 31.71%). Across multiple BMI levels and sexes, there were significant variations in MetS prevalence between several Asian ethnic groups and their NHW counterparts, as demonstrated by the non-overlapping 95% CIs across ethnic groups shown in Fig. 1.

There were also some notable gender differences. Among individuals with a BMI < 27.5 (normal or overweight, i.e., non-obese) across all Asian ethnic groups, MetS prevalence was higher in women than in men, with an average of 21.38% in women versus 5.94% in men for BMI < 23, and 49.37% in women versus 37.69% in men for BMI between 23 and 27.4. For those with a BMI ≥ 27.5, prevalence rates were similar across genders, with men generally exhibiting slightly higher rates than women, except in the Korean subsample. We also observed differences in how MetS prevalence varied by BMI level between sexes. In the Asian Indian subsample, for example, MetS prevalence among women was more clustered across BMI levels, as evidenced by overlapping confidence intervals and a range of less than 30% points (33.44–62.84%). In contrast, prevalence among men spanned a broader range, with a significant difference of nearly 70% points across BMI levels (8.58–76.56%). In other words, the prevalence of MetS among women varied less by BMI, while there was a greater variation among men. This pattern also held for NHW and all five Asian ethnic groups except for Koreans. Detailed sociodemographic and lifestyle characteristics of the aggregate Asian American sample from this dataset have been previously published^48^.

Table 1 presents the log odds and discrete change of binary logistic regression results of sociodemographic and lifestyle behaviors on MetS in each of the five Asian ethnic groups. All estimates were mutually adjusted for all other variables in the model. In all five models, BMI and age were positively associated with MetS. Specifically in the Chinese and Korean sub-samples, a non-linear (quadratic) relationship was observed for BMI, with a positive main effect indicating increased MetS risk at lower to moderate BMI levels, followed by a tapering or slight decline in risk at higher BMI levels. This pattern reflects an inverted U-shaped relationship between BMI and MetS, where MetS risk initially increases with BMI before leveling off and decreasing slightly as BMI continues to rise. A similar curvilinear relationship was observed between age and MetS in the Korean subsample, with the quadratic term of age being marginally significant (*p* <.10).

**Table 1:** Results of multivariate logistic regressions of sociodemographic and lifestyle behaviors on MetS in five Asian American ethnic groups: NHANES 2011–2016 (*N* = 1,781).

		Chinese (*n* = 652)		Indian (*n* = 409)		Filipino (*n* = 262)	
		log odds (95% CI)	discrete change	log odds (95% CI)	discrete change	log odds (95% CI)	discrete change
BMI		1.9115*** (0.9269, 2.8961)	0.0554***	0.8515* (0.1273, 1.5757)	0.0476***	0.9784* (0.0926, 1.8643)	0.0517***
BMI squared		−0.0286** (‐0.0473, ‐0.0099)		−0.0110† (‐0.0238, 0.0018)		−0.0106 (‐0.0267, 0.0055)	
age		0.0927 (−0.0372, 0.2226)	0.0095***	0.0723 (−0.0817, 0.2263)	0.0086***	0.0542 (−0.1181, 0.2264)	0.0111***
age squared		−0.0002 (‐0.0015, 0.0010)		−0.0002 (‐0.0018, 0.0014)		0.0004 (−0.0013, 0.0021)	
women (ref: men)		0.6941* (0.0517, 1.3365)	0.0916*	−0.0623 (‐0.6886, 0.5640)	−0.0100	0.1945 (−0.7771, 1.1662)	0.0218
currently married (ref: not)		−0.4096 (‐1.1242, 0.3050)	−0.0540	0.5250 (−0.4218, 1.4719)	0.0856	0.2104 (, −0.9194, 1.3402)	0.0247
education (ref: < high school)							
high school		0.1227 (−0.6394, 0.8848)	0.0162	0.1120 (−1.2831, 1.5070)	0.0189	−0.4099 (‐1.8975, 1.0776)	− 0.0487
college or higher		−0.0945 (‐0.7908, 0.6019)	−0.0128	−0.6570 (‐1.9750, 0.6609)	−0.1070	−0.6052 (‐2.0244, 0.8141)	− 0.0715
ratio of family income to poverty		0.0112 (−0.1490, 0.1714)	0.0016	0.0813 (−0.1087, 0.2712)	0.0130	0.0903 (−0.2886, 0.4691)	0.0100
physical activity (ref: sedentary)							
insufficient		−0.0756 (‐0.7955, 0.6442)	−0.0101	−0.1118 (‐0.9716, 0.7480)	−0.0182	−0.1885 (‐1.4212, 1.0443)	− 0.0235
moderate		−0.3961 (‐1.2392, 0.4469)	−0.0528	−0.5929 (‐1.6040, 0.4181)	−0.0960	−0.7315 (‐2.5176, 1.0546)	− 0.0900
high		−0.6914 (‐1.5296, 0.1467)	−0.0906	−0.4634 (‐1.5074, 0.5806)	−0.0754	−1.3855 (‐2.7960, 0.0251)	− 0.1638
current smoker (ref: not)		0.35185 (−0.6780, 1.3817)	0.0476	0.16492 (−0.9989, 1.3288)	0.0269	0.6351 (−1.2216, 2.4918)†	0.0736†
alcohol consumption (ref: lifetime abstainer)							
former drinker		−0.3856 (‐1.3213, 0.5501)	−0.0481	0.3382 (−0.8148, 1.4912)	0.0542	−1.1097 (‐2.9580, 0.73855)	− 0.1208
non-excessive current drinker		0.25704 (−0.5177, 1.0318)	0.0332	0.1659 (−0.5962, 0.9279)	0.0267	−0.6746 (‐1.6554, 0.3061)	− 0.0814
excessive current drinker		0.3723 (−1.2269, 1.9714)	0.0488	0.3815 (−1.0040, 1.7670)	0.0617	−0.7927 (‐3.5226, 1.9373)	− 0.0938
constant		−34.1436		−17.5631		−20.8453	
		Vietnamese (*n* = 243)		Korean (*n* = 215)			
		log odds (95% CI)	discrete change	log odds (95% CI)	discrete change		
BMI		2.4991* (0.0575, 4.9408)	0.0582***	1.6390** (0.7502, 2.5279)	0.0672***		
BMI squared		−0.0375 (‐0.0845, 0.0094)		−0.0209** (‐0.0337, ‐0.0080)			
age		0.1216 (−0.0645, 0.3076)	0.0069**	0.2269 (−0.0136, 0.4675)	0.0077**		
age squared		−0.0006 (‐0.0024, 0.0011)		−0.0017† (0.004, 0.0006)			
women (ref: men)		1.2025 (0.1645, 2.2405)	0.1360*	1.1358† (−0.1750, 2.4466)	0.1260†		
currently married (ref: not)		0.1417 (−0.7490, 1.0324)	0.0158	−0.1667 (‐1.4260, 1.093)	− 0.0192		
education (ref: < high school)							
high school		0.0463 (−1.1533, 1.2459)	0.0054	−1.0250 (‐3.7946, 1.7447)	0.1153		
college or higher		−0.7291 (‐2.4883, 1.0300)	−0.0826	−0.8483 (‐3.5011, 1.8044)	− 0.0938		
ratio of family income to poverty		−0.0830 (‐0.4461, 0.2802)	−0.0093	−0.0245 (‐0.34925, 0.3002)	− 0.0027		
physical activity (ref: sedentary)							
insufficient		0.1125 (−0.9417, 1.1667)	0.0123	−0.2949 (‐2.0177, 1.4278)	− 0.0346		
moderate		0.4165 (−0.9574, 1.7903)	0.0482	−0.8870(‐2.5576, 0.7836)	− 0.1023		
high		−0.6409 (‐2.4381, 1.1563)	−0.0684	−1.7921† (‐3.9465, 0.3624)	− 0.0241†		
current smoker (ref: not)		0.0422 (−1.4697, 1.5541)	0.0042	0.4497 (−1.0499, 1.9493)	0.0518		
alcohol consumption (ref: lifetime abstainer)							
former drinker		0.5040 (−1.1407, 2.1488)	0.0573	0.5217 (−2.3740, 3.4173)	0.0561		
non-excessive current drinker		0.2048 (−0.9610, 1.3706)	0218	0.1870 (−1.8106, 2.1845)	0.0183		
excessive current drinker		0.6173 (−1.6046, 2.8392)	0.0706	1.4266 (−1.7171, 4.5703)	0.1581		
constant		−44.1941		−33.5644			

Abbreviation: MetS = metabolic syndrome; NHANES = National Health and Nutrition Examination Survey; CI = confidence interval.† *p* <.1; * *p* <.05; ** *p* <.01; *** *p* <.001.

In addition, after controlling for age, BMI, marital status, education, poverty ratio, physical activity, smoking, and drinking, we found that Chinese and Vietnamese women were significantly more likely to have MetS than their male counterparts (*p* <.05), by 9.16 and 13.60% points, respectively. A similar trend was observed in the Korean subsample, though this sex difference was only marginally significant (*p* <.10).

Two lifestyle behaviors (smoking and physical activity) had marginally significant associations with MetS risk. Specifically, Filipino Americans who were current smokers showed a marginally higher likelihood of having MetS compared to their non-smoking counterparts (*p* <.10), with other covariates held constant. Korean Americans with high levels of physical activity were marginally less likely to have MetS than those with a sedentary lifestyle (*p* <.10). Alcohol consumption was not statistically significant.

In summary, BMI, age, and gender appeared consistently associated with a higher odds of MetS across the five groups, with marginal associations observed for certain lifestyle behaviors in particular groups. Marital status, education, poverty level, and alcohol consumption were included in the analysis but did not show significant associations with MetS in any ethnic group.

**Fig. 2:**
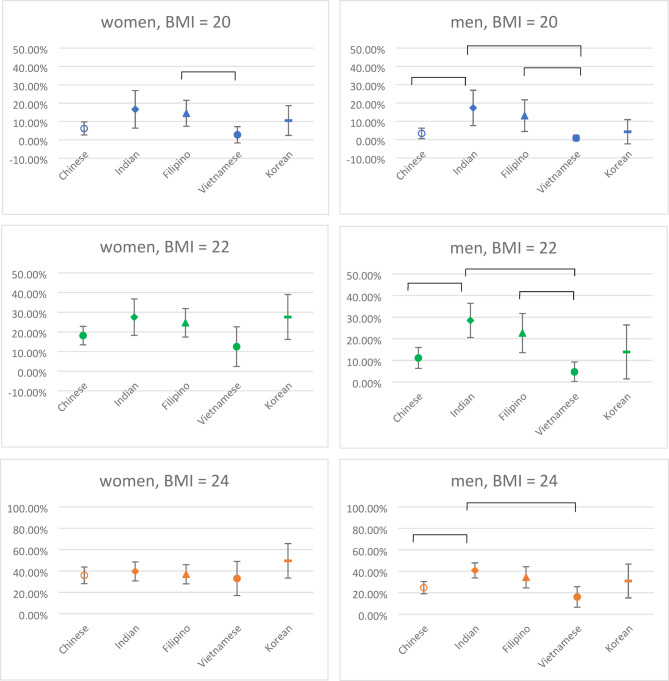
Predicted Probabilities and 95% CI of MetS by BMI (20, 22, and 24) and Sex Based on Logistic Regression Results: NHANES 2011–2016 (*N* = 1,781). Note: The error bars represent 95% confidence intervals. Brackets indicate statistically significant differences between ethnic groups (*p* <.05).

The scatter plots in Fig. 2 show the predicted probabilities of MetS by BMI (20, 22, and 24) and sex. Predicted probabilities of MetS were calculated based on the logistic regression results, with all other covariates held at their sample mean values. Among women, significant differences in predicted probabilities of MetS were observed between Filipino and Vietnamese women at a BMI of 20, with Filipino women (14.46%, 95% CI: 7.36% − 21.57%) showing a significantly higher predicted probability than their Vietnamese counterpart (2.73%, 95% CI: -1.72% − 7.18%). Among men, there were more ethnic differences; at a BMI of 20, Indian men (17.32%, 95% CI: 7.65% − 26.99%) had significantly higher predicted MetS risk compared to their Chinese (3.40%, 95% CI: 0.52% − 6.28%) and Vietnamese (0.86%, 95% CI: − 0.69% − 2.41%) counterparts, and Filipino men (13.08%, 95% CI: 4.45% − 21.71%) also had significantly higher risks than Vietnamese men. This pattern persisted at BMI 22. At BMI 24, considered overweight, Indian men (40.79%, 95% CI: 33.78% − 47.81%) continued to show a significantly higher risk of MetS compared to both Chinese (24.72%, 95% CI: 19.03% − 30.42%) and Vietnamese men (16.07%, 95% CI: 6.50% − 25.64%). In summary, after adjusting for covariates, Indian and Filipino men exhibited significantly higher MetS risks than their Chinese and Vietnamese counterparts across BMI levels 20 to 24, with this ethnic disparity being especially pronounced in men.

There were no significant differences in predicted probability of MetS found across Asian ethnic groups for either men or women with BMI levels other than 20, 22, and 24. Thus the predicted probabilities for BMI levels other than 20, 22, and 24 were not reported in this article. Results for these additional BMI levels are available upon request.

## Discussion

The findings of this nationally representative study provide valuable insights into the complex relationships between BMI, ethnicity, and MetS risk among various Asian American subpopulations. Several key points emerged from our analysis. First, we found significant heterogeneity in MetS risk across Asian American subpopulations; this heterogeneity was most pronounced when examining MetS prevalence across BMI levels and sex. For example, among those with a BMI < 23 (normal), MetS prevalence was significantly higher among Filipina compared to Chinese women. Among men with a BMI between 23 and 27.4, MetS prevalence was significantly higher among Asian Indian compared to Vietnamese men. Prior studies suggest a higher MetS prevalence among Asian Americans than NHW at lower BMI categories^49^, and our disaggregated analyses unmask potential ethnic differences, showing that certain Asian American groups exhibit greater MetS prevalence at lower levels of BMI. Disaggregating Asian American health data is critical to addressing the harms of treating this group as monolithic, as differences in MetS burden may worsen CVD disparities. Our findings also highlight the need for targeted approaches, such as sex- and subpopulation-specific screening for high-risk groups like Filipino women and Asian Indian men, who face elevated MetS risk at lower BMIs. This pattern is consistent with emerging research on ethnic-specific metabolic profiles and aligns with studies demonstrating variable cardiometabolic risk thresholds across Asian populations^50–54^.

Most Asian American subgroups showed higher MetS prevalence in women than men at normal-to-overweight BMI levels. In obesity, MetS rates were similar between genders, with men slightly higher—except among Koreans, who showed no sex difference. Korean women also exhibited greater variation in MetS prevalence by BMI compared to men, unlike other subpopulations. This suggests unique genetic, lifestyle, or environmental influences on MetS risk in Korean women, reflecting the broader genetic, dietary, and cultural diversity across Asian ethnic groups and sexes that necessitates tailored approaches to prevention, screening, clinical assessment, and treatment^55–57^.

This study highlights key differences in MetS prevalence across Asian American subpopulations. Given MetS’s strong association with cardiometabolic diseases - including type 2 diabetes, CAD, and stroke - targeted interventions are crucial to improve health outcomes among high-risk populations. Recent disaggregated national data further confirms these subpopulation variations in cardiometabolic disease prevalence. National data from 2013 to 2021 on seven Asian American subpopulations found that the cardiometabolic disease risk was higher among Filipinos (twice as likely to have CAD, previous heart attack, and stroke) and Asian Indians (1.5 times more likely to have hypertension and hyperlipidemia) compared to the other groups studied^58^. National Health Interview Survey (NHIS) data showed that diabetes was highest among Asian Indians, while hyperlipidemia, hypertension, and obesity were highest among Filipinos, and Chinese had the lowest odds of CVD risk factors assessed^10,58^. Therefore, evidence-based clinical decision-making and public health efforts should be in line with addressing MetS as a risk factor for these noted cardiovascular health differences to prevent further morbidity and mortality from CVD among certain subpopulations^59,60^.

Our findings also shed light on the nuanced associations between modifiable lifestyle factors and MetS risks in these Asian subpopulations. Specifically, we found that smoking was associated with a marginally higher MetS prevalence in Filipinos. In comparison, high levels of physical activity were linked to a marginally lower MetS prevalence in Koreans. While previous studies have illustrated that lower physical activity is associated with greater MetS among Asian Americans as an aggregate^61^, few to no studies examine lifestyle behaviors and their associations with MetS among disaggregated groups and have primarily examined how they are associated with individual components of MetS. These findings provide potential insights into understanding how certain lifestyle factors may uniquely influence specific ethnic groups experiencing MetS, opening the door for the potential for targeted lifestyle interventions to reduce MetS risk in specific ethnic groups.

Our study has some limitations. The cross-sectional nature of the data limits causal inferences. Additionally, while we examined five Asian ethnic subgroups, the sample sizes for some groups were relatively small, potentially limiting the generalizability of our findings. Future longitudinal studies with larger, more diverse samples are needed to elucidate further the complex relationships between ethnicity, BMI, gender, lifestyle factors, and MetS risk. Including more Asian American subgroups not captured or well-represented currently in NHANES would allow us to characterize further MetS risk among these populations and potential racial and ethnic disparities in cardiovascular health. Additionally, this study did not distinguish between underweight and normal weight BMI categories, which may have obscured potential differences in MetS prevalence between these groups, particularly given that underweight status may be associated with different health risks in some Asian American populations.

Our findings reveal significant heterogeneity in MetS burden across Asian American subpopulations, underscoring the need for subpopulation-specific prevention, screening, and management strategies. Public health efforts must account for subpopulation differences in sex, BMI, and lifestyle factors. As the Asian American population grows, addressing these differences will be critical to reducing cardiovascular disease burden. Tailored approaches by healthcare providers and policymakers can help mitigate MetS risk and its complications in these communities.

## Supplementary Information

Below is the link to the electronic supplementary material.

Supplementary Material 1

## Data Availability

The datasets analyzed in this study, NHANES 2011–2016, are available on the website of the National Center for Health Statistics, https://wwwn.cdc.gov/nchs/nhanes/Default.aspx, accessed on 26 October 2018.
