# Supplementary material for: Distinct metabolic syndrome profiles across Asian American subpopulations
Source: Sci Rep. 2025 Aug 12;15:29518. doi: 10.1038/s41598-025-15183-6 (PMC12343788; doi:10.1038/s41598-025-15183-6)
Supplement: Supplementary file 1 — Supplementary Material 1 [file 41598_2025_15183_MOESM1_ESM.docx]

**Supplementary Materials**

| Table S1. MetS as defined by IDF criteria | |
| --- | --- |
| CRITERIA: Central obesity (defined as waist circumference ≥ 90 cm for South and East Asian men and ≥ 80 cm for South and East Asian women, with ethnicity specific values, assumed if BMI is > 30 kg/m^2^) | |
| plus, any two of the following four factors: | |
| Raised triglycerides | ≥ 150 mg/dL |
|  | or specific treatment for this lipid abnormality |
| Reduced HDL cholesterol | < 40 mg/dL in males  < 50 mg/dL in females |
|  | or specific treatment for this lipid abnormality |
| Raised blood pressure | ≥ 130/85 mm Hg |
|  | or treatment of previously identified hypertension |
| Raised fasting plasma glucose | ≥ 100 mg/dL |
|  | or previously diagnosed T2DM |
| Abbreviation: MetS = metabolic syndrome; IDF = International Diabetes Federation; BMI = body mass index; HDL = high-density lipoproteins; T2DM = type 2 diabetes mellitus | |

| **Table S2. Age-Adjusted, Sex-Specific Prevalence of MetS and 95% CI at Three BMI Levels among Five Major Asian American Ethnic Groups and Non-Hispanic Whites: NHANES 2011–2016 (N = 8,099)** | | | | | | |
| --- | --- | --- | --- | --- | --- | --- |
| BMI level | Chinese | Asian Indian | Filipino | Vietnamese | Korean | NHW |
| women | % (95% CI) | | | | | |
| < 23 | 14.45  (10.08– 18.82) | 33.44  (18.62– 48.26) | 31.70  (19.92– 43.47) | 13.53  (3.87– 23.19) | 13.77  (3.56– 23.98) | 16.47  (12.48– 20.46) |
| 23 – 27.4 | 46.46  (35.13– 57.79) | 59.26  (48.92– 69.61) | 43.66  (29.31– 58.01) | 43.49  (30.81– 56.17) | 53.96  (38.52– 69.41) | 34.44  (30.01– 38.87) |
| >= 27.5 | 65.97  (47.30– 84.63) | 62.84  (49.14– 76.52) | 72.27  (55.20– 89.33) | 74.11  (56.16– 92.06) | 89.63  (72.92– 106.35) | 62.95  (59.83– 66.07) |
| men | % (95% CI) | | | | | |
| < 23 | 3.95  (-1.09– 8.90) | 8.58  (-1.06– 18.22) | 5.44  (-3.34– 14.29) | 7.84  (-1.02– 16.71) | 3.89  (-2.00–9.78) | 10.24  (7.03–13.46) |
| 23 – 27.4 | 36.28  (28.45– 44.11) | 50.80  (42.64– 58.96) | 34.20  (24.47– 43.92) | 22.66  (13.60– 31.71) | 44.51  (28.79– 60.23) | 42.72  (39.07– 46.38) |
| >= 27.5 | 69.70  (58.53– 80.88) | 76.56  (65.49– 87.63) | 77.15  (60.13– 94.17) | 81.80  (48.65– 114.96) | 74.34  (49.04– 99.64) | 71.64  (68.68– 74.59) |
| Abbreviation: MetS = metabolic syndrome; CI = confidence interval; NHANES = National Health and Nutrition Examination Survey; BMI – body mass index; NHW = non-Hispanic white | | | | | | |
